# Effect of sodium fluorescein strip application on cornea parameters commonly used in laser-assisted in-situ keratomileusis

**DOI:** 10.1038/s41598-023-46502-4

**Published:** 2023-11-04

**Authors:** Dongmei Han, Wenjuan Xie, Muqu Yuan, Jing Cui, Qifeng Wang, Qingsong Zhang

**Affiliations:** Aier Eye Hospital of Wuhan University (Wuhan Aier Eye Hospital), Wuhan, China

**Keywords:** Diseases, Medical research

## Abstract

To assess the effect of sodium fluorescein (NaF) strip on corneal parameters commonly used in Laser-assisted in-situ keratomileusis (LASIK). Eighty-six subjects (172 eyes) scheduled for LASIK were recruited between January and March 2022. The study and statistical analysis test were conducted in April 2022. Topographic measurements of corneal parameters, including central corneal thickness (CCT), anterior keratometric (K) readings (K1, flat keratometry; K2, steep keratometry), horizontal corneal diameter (white to white, WTW), and corneal asphericity (Q value), were obtained using a Scheimpflug device (Pentacam) before and 10 min after NaF strip treatmentThe Pentacam recorded a small significant increase in CCT (mean 538.88 ± 28.78 μm to 547.90 ± 29.94 μm; *p* < .001), with no differences in K1 and K2 (mean 42.24 ± 1.35D to 42.24 ± 1.35D, and mean 43.34 ± 1.50D to 43.32 ± 1.51D; *P* > .05, for all) as well as WTW(mean 11.58 ± 0.32 mm to 11.58 ± 0.32 mm, *P* > .05) before and after NaF strip intervention. Furthermore, there was no significant difference was observed in Q value (mean − 0.30 ± 0.13 to − 0.30 ± 0.14, *P* > .05). These results indicate that clinicians should avoid NaF strip application before obtaining precise topographic measurements of cornea parameters using the Pentacam.

## Introduction

Laser-assisted in-situ keratomileusis (LASIK) is currently the most popular laser refractive surgery technique with its good visual outcomes, rapid post-operative recovery and good safety profile^1^. The Accurate measurement of corneal parameters is essential in determining the suitability and preoperative planning of patients for LASIK. Central corneal thickness (CCT) and anterior keratometric (K) readings (K1, flat keratometry; K2, steep keratometry) are the main determining factors of excimer laser ablation^2^. Furthermore, the measurements of horizontal corneal diameter (white to white, WTW) and corneal asphericity (Q value) are also important in LASIK.

A healthy and structurally intact corneal epithelium is considered a prerequisite for corneal refractive surgery, including LASIK, which is known to cause or exacerbate dry eye symptoms and corneal epithelium defects, resulting in fluctuating vision, and unavoidably lowering patient satisfaction. Therefore, preoperative detection of the corneal epithelium and screening for dry eye are of most importance in refractive surgery. Sodium fluorescein (NaF) (weight 376.67 dalton), is a weak dibasic acid^3^. NaF, delivered as a 0.25% solution or via a paper strip, has been clinically applied to detect corneal epithelial abrasions in various forms of punctate keratitis or epithelial defects, as well as to examine the status of the precorneal tear film with respect to tear break-up (BUT)^4^. Until recently, NaF intervention has remained a key step in evaluating corneal epithelial defects and diagnosing dry eye before corneal refractive surgery.

The Pentacam (Oculus Optikgeräte GmbH, Wetzlar, Germany) is one of the most commonly used corneal tomographic technologies clinically, which consists of a rotating Scheimpflug camera and a monochromatic slit light source (blue led at 475 nm) that rotates together around the optical axes of the eye to calculate a three-dimensional model of the anterior segment^5^, including corneal parameters such as CCT, K1, K2, and Q value. Prior reports have demonstrated the excellent repeatability of measurements obtained with the Pentacam in healthy eyes^6^.

Although the effect of NaF on central and peripheral corneal thickness, as well as other anterior segment parameters, such as anterior chamber volume, iridocorneal angle, limbus-limbus distance, and corneal volume have been investigated, little is known about the effect of NaF strip application on corneal parameters commonly used for LASIK. Therefore, this study aimed to investigate the effect of the NaF strip on corneal parameters obtained with the Pentacam to provide precise measurements of corneaparameters for LASIK.

## Results

### Average measurements and normal distributions of cornea parameters

A total of 172 eyes from 86 subjects scheduled for LASIK were recruited for this study. The mean age of the participants was 22.91 ± 4.64 years (range 17–34 years), and 52 (60.47%) were female. Table 1 shows the average CCT, K1, K2, WTW, and Q valuebefore and 10 min after treatment with NaF strip, as well as the normal distributions of all cornea parameters. Before NaF strip intervention, the average measurements of CCT, K1, K2, WTW, and Q value were 538.88 ± 28.78 μm, 42.24 ± 1.35 D, 43.34 ± 1.50 D, 11.58 ± 0.32 mm, and − 0.30 ± 0.13, respectively. After 10 min of treatment with NaF strip, the average values of the same parameters were 547.90 ± 29.94 μm, 42.24 ± 1.35 D, 43.32 ± 1.51 D, 11.58 ± 0.32 mm, and − 0.30 ± 0.14, respectively. According to the one-sample Kolmogorov–Smirnov test, the measurments of CCT, NaF CCT, Q, and NaF Q valueall exhibited normal distributions (P > 0.05, for all). In contrast, the values of K1, NaF K1, K2, NaF K2, WTW, and NaF WTW all exhibited non-normal distributions (P < 0.05, for all).

**Table 1:** Average measurements and normal distributions of cornea parameters.

One-sample Kolmogorov–Smirnov Test										
	CCT (μm)	KI (D)	K2 (D)	WTW (mm)	Q value	NaF CCT (μm)	NaF KI (D)	NaF K2 (D)	NaF WTW (mm)	NaF Q value
N	172	172	172	172	172	172	172	172	172	172
Mean	538.88	42.24	43.34	11.58	− 0.30	547.90	42.24	43.32	11.58	− 0.30
SD	28.78	1.35	1.50	0.32	0.13	29.94	1.35	1.51	0.32	0.14
Asymp. Sig. (2-tailed)	0.200	0.000	0.014	0.014	0.200	0.200	0.000	0.000	0.018	0.200

*CCT* central corneal thickness; K1, flat keratometry; K2, steep keratometry, *WTW* horizontal corneal diameter, white to white; Q value, cornea asphericity, *NaF* sodium fluorescein. The statistical significance level was set at *P* < .05.

### The effect of NaF strip application on CCT

**Figure 1:**
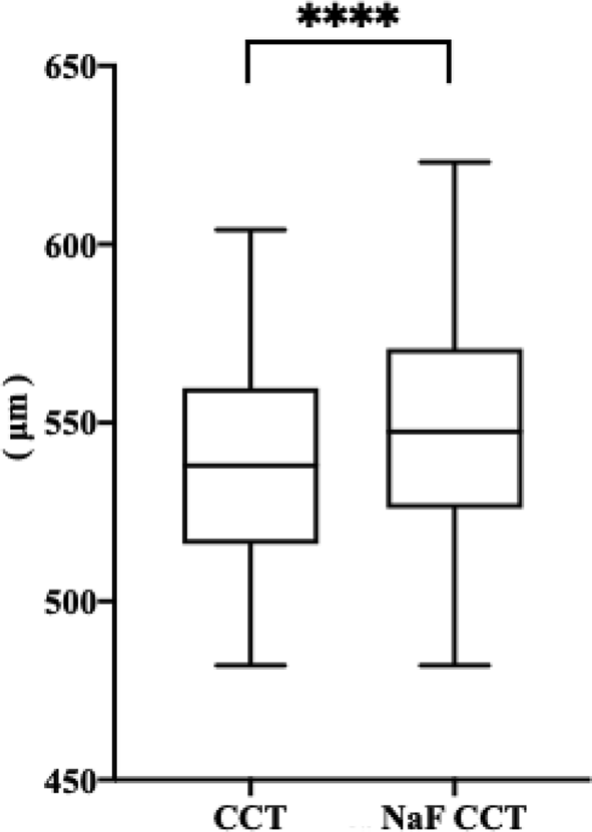
Box plot shows the difference between the measurements of CCT and NaF CCT. CCT, central corneal thickness; NaF, sodium fluorescein. ****P < .001.

Figure 1 shows the differences between CCT measurements before and 10 min after NaF strip intervention obtained with the Pentacam. Before NaF strip treatment, the average value of CCT was 538.88 ± 28.78 μm. After NaF strip application, however, the average measurement of CCT significantly increased to 547.90 ± 29.94 μm (△ = 9.02 ± 8.33 μm). In addition, according to the 1-sample Kolmogorov–Smirnov test, CCT and NaF CCT measurements all exhibited normal distributions (*P* > 0.05, for all). Thus, a paired samples t-test was used to investigate the difference between these two values, with the results indicateda statistically significant difference (t = − 14.20, *P* < 0.001).

### The effect of NaF strip application on K readings

**Figure 2:**
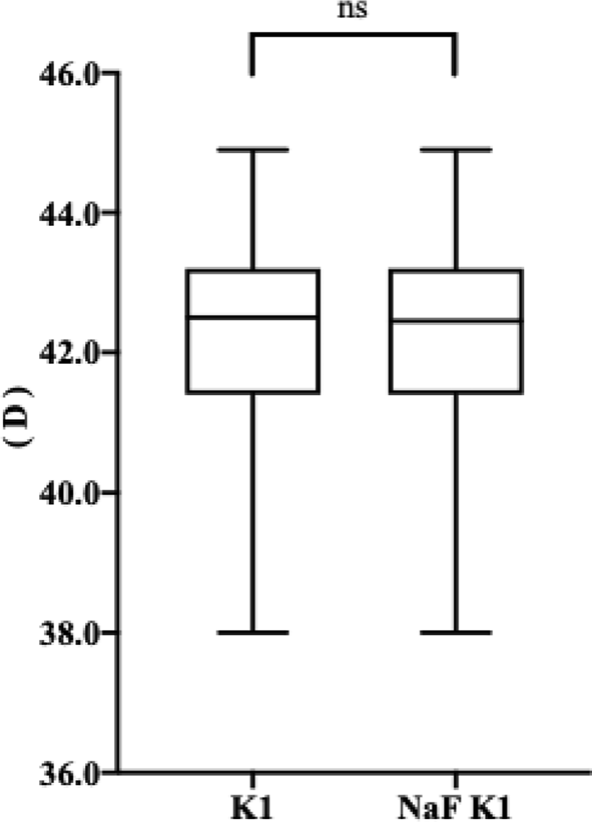
Box plot shows the difference between the measurements of KI and NaF K1 at diameters of 4 mm. K1, flat keratometry; NaF, sodium fluorescein. ns, no statistically significance.

Figure 2 shows the difference between the measurements of K1 at diameters of 4 mm before and 10 min after treatment with NaF strip using the Pentacam. Before NaF strip intervention, the average value of K1 was 42.24 ± 1.35 D. After NaF strip application, this value was 42.24 ± 1.35D (△ = 0.00 ± 0.16). According to the 1-sample Kolmogorov–Smirnov test, the measurements of K1 and NaF K1 all exhibited non-normal distributions (*P* < 0.05, for all), and the Wilcoxon signed-rank test was therefore used to compare the difference between these two measurements, with the results showed no statistical significance (Z = − 0.005, *P* = 0.996).

**Figure 3:**
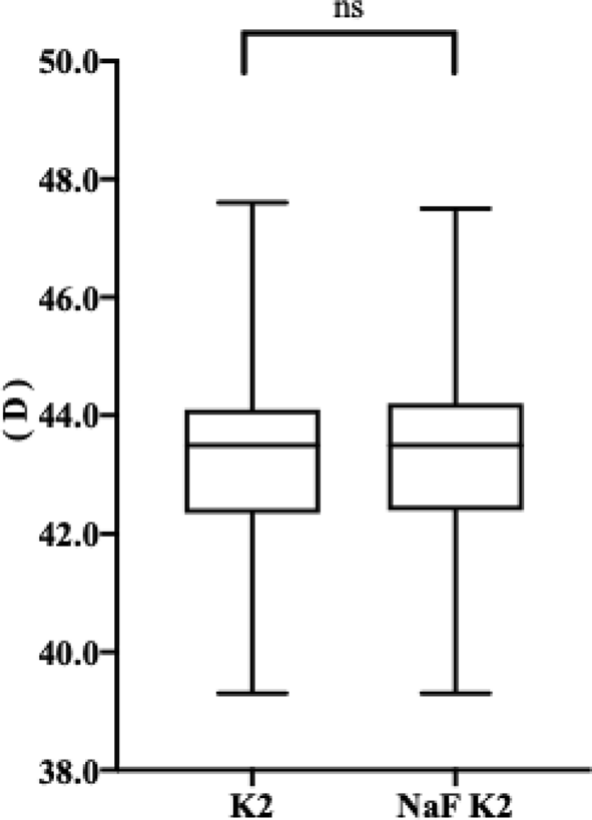
Box plot shows the difference between the measurements of K2 and NaF K2. K2, steep keratometry; NaF, sodium fluorescein. ns, no statistically significance.

Figure 3 shows the difference between the measurements of K2 at a diameter of 4 mm before and 10 min after NaF strip application using the Pentacam. Before NaF strip intervention, the average value of K2 was 43.34 ± 1.50 D on average. After treatment with the NaF strip, the average value of K2 was 43.32 ± 1.51 D (△ = 0.01 ± 0.25). Moreover, according to the 1-sample Kolmogorov–Smirnov test, the measurements of K2 and NaF K2 all exhibited non-normal distributions (*P* < 0.05, for all), and the Wilcoxon signed-rank test was used to compare the difference between these two values. No statistically significant differences were detected (Z = − 1.520, *P* = 0.129).

### The effect of NaF strip application on WTW

**Figure 4:**
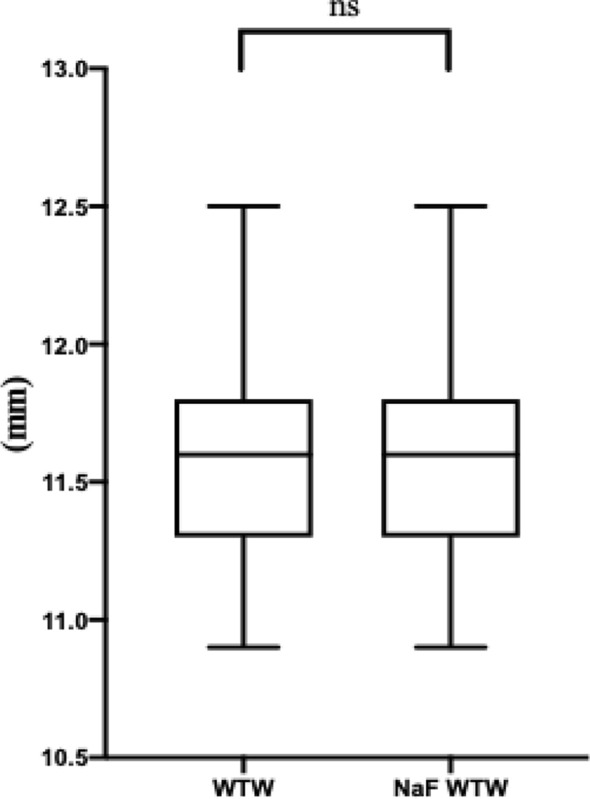
Box plot shows the difference between the measurements of WTW and NaF WTW. WTW, white to white; *NaF* sodium fluorescein. *ns* no statistically significance.

Figure 4 shows the difference between the WTW measurements before and 10 min after NaF strip intervention obtained with the Pentacam. Before NaF strip application, the average WTW was 11.58 ± 0.32 mm. After 10 minof NaF strip treatment, this value was 11.58 ± 0.32 mm (△ = 0.00 ± 0.07). According to the 1-sample Kolmogorov–Smirnov test, the measurements of WTW and NaF WTW all exhibited non-normal distributions (*P* < 0.05, for all), and the Wilcoxon signed-rank test was therefore used to compare the difference between these two measurements. No statistically significant difference was observed (Z = − 0.582, *P* = 0.561).

### The effect of NaF strip application on Q value

**Figure 5:**
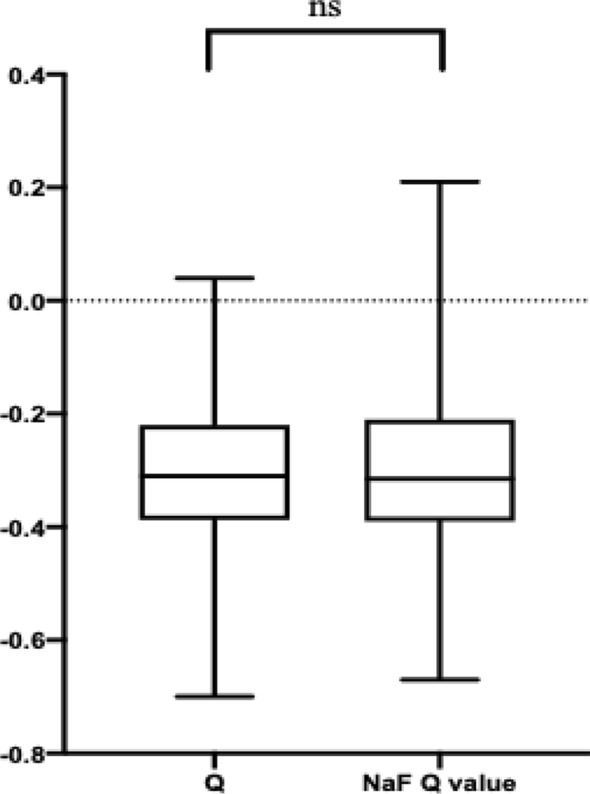
Box plot showshe difference between the measurements of Q value and NaF Q value. Q value, cornea asphericity; *NaF* sodium. fluorescein. *ns* no statistically significance.

Figure 5 shows the difference between the measurements of Q value at diameters of 8 mm before and 10 min after treatment with NaF strip by the Pentacam. Before treatment with NaF strip, the average Q value was − 0.30 ± 0.13, while this value was − 0.30 ± 0.14 (△ = 0.00 ± 0.06) after intervention. According to the 1-sample Kolmogorov–Smirnov test, the Q and NaF Q values exhibited normal distributions (*P* > 0.05, for all); thus, a paired samples t-test was used to investigate the difference between these two measurements, with the results indicated no statistically significant differences (t = − 0.482, *P* = 0.630).

## Discussion

To the best of our knowledge, this study is the first to report the effectof NaF strip intervention on corneal parameters commonly used in LASIK. CCT, K1, K2, WTW, and Q value are all related to the shape of cornea, and are thus of utmost importance in LASIK.

Cornea refractive surgeons have relied on the estimation of CCT reduction on the laser platform to calculate the thickness of the residual stromal bed after surgery, thereby minimizing the risk of corneal keratectasia after surgery^7,8^. In addition, CCT have been relied on to design the ablation zone diameters, which is closely related to postoperative visual quality. For example, a smaller ablation zone diameter normally results in an increase corneal HOAs^9^, which is associated with lower visual quality postoperatively. In our study, an apparent increase in CCT was found, and this increase showed the potential significant errors in preoperative planning, which is not only concerned screening the risk of postoperative corneal keratectasia, but also the evaluation of postoperative visual quality for LASIK^10^. The precise measurement of corneal power has progressively gained importance in parallel with the development of refractive surgery procedures^11,12^. We chose the K readings at the 4 mm zone in this study as previous studies have shown that this was the most accurate zone of measurements^10,13^. In LASIK, the appropriate suction ring is selected mainly depend on the corneal steepness. In addition, abnormal findings at preoperative examination using the Pentacam, such as inferior steepening is one of important factors in developing postoperative ectasia^14,15^. Our study observed that NaF strip intervention did not cause a statistically significant change in K readings^15^, including K1 and K2. We this concluded that refractive surgeon need not take this change into account in selecting the suction ring and screening preoperative keratoconus or postoperative corneal ectasia in LASIK.

Mustafa Dog ˘an et al. previously reported that the limbus-limbus distance (LLD) values at the 1st, 5th, 15th, and 30th minute after application of the fluorescein strip were not significantly changed compared to the pre-application values^16^. This result is in agreement with our findings. In femtosecond laser-assisted laser in situ keratomileusis (FS-LASIK), the formation of anterior chamber gas bubbles during flap creation with a femtosecond laser is not an uncommon event, especially in Asian eyes, which typically have a small WTW corneal diameter^17^. In addition, the appropriate suction ring is selected also dependon the corneal diameter in LASIK. Our study indicated that the effect of NaF strip treatment on the measurement of WTW was too small to take into account.

Corneal asphericity at the anterior corneal surface is negative and increases from the center to the periphery^18^. Esraa El-Mayah et al. previously suggested that the spherical aberration at 6.0 mm is affected by the ablation profile method, but the Qvalue at 8.0 mm is a parameter that could predict ablation volume or ablation depth relatively independently of ablation profile^19^; as such, the measurement of the Qvalue in the 8 mm zone was investigated in our study. In LASIK, monovision is a method of presbyopic correction, in which one eye is corrected for distance vision and the other eye for near vision, and this procedure is based on Q factor modulation. In addition, the Q factor was adjusted in both eyes, which induced an increase in the negative Q factor and improved the prolate shape (hyperprolateness) of the cornea^20^, and using Q-optimized algorithms improves visual quality for myopia in terms of the modulation transfer function (MTF)^21,22^. Our analysis showed that NaF strip application did not cause a statistically significant change in the measurement of Q value; therefore, we considered that NaF strip intervention would not significantly influence the preoperative planning in LASIK with a custom Q-ablation profile.

To sum up, our investigation showed that the prominent influence of NaF strip intervention on corneal parameters normally used in LASIK was restricted to CCT, and the effect on other corneal parameters, such as K readings, WTW, and Q value, was too small to be considered in preoperative planning and postoperative evaluation. Some studies have previously investigated the effect of the NaF strip and anesthetic substance + NaF mixture on CCT. Mustafa Dog ˘an et al. reported that the mean CCT before NaF strip application was 552.83 ± 36.51 μm, rising to 556.68 ± 36.73 μm at the 1st minute^16^. Briggs et al. reported that when they applied a group NaF 2% and another group of saline drops, a mean increase of 37.0 μm in CCT at the 1st minute in the NaF-infused group^23^. Ditipriya et al. showed that ultrasound pachymetry combined with proparacaine 0.50%–NaF 0.25% caused a small (< 10 mm) but significant level of corneal swelling.^24^. Moreover, Zhuang et al. applied a 0.1% drop of NaF and measured CCT with the Pentacam, and found that the mean central tear film thickness in normal eyes was 24.7 ± 3.9 μm (range, ~ 17–32 μm), while the premeasurement difference was lower in patients with dysfunctional tear syndrome than in healthy volunteers. They attributed this to the tear film layer^25^. Firstly, it needs to be emphasized that the different devices use the different examination patterns, which result in the different measurement rusults. Secondly, in our study, we detected an average increase of 9.02 ± 8.33 μm in the CCT at 10 min after NaF strip treatment, however, which may be inappropriate explained by corneal swelling, for it is usually caused by anesthetic substance. Moreover, we considered it reasonable to partially attribute this increase to the thickening of the tear film layer after NaF strip treatment. Last but not least, the Pentacam uses a rotating Scheimpflug camera and a UV-free 475 nm blue slit LED light source. The increase in the measurement of CCT observed in this study may be attributed to the intense yellow-green NaF on the corneal surface, which forms a high-density reflection. As soon as the camera captured the photographs, this reflection resulted in the incorrect recognition of the precorneal boundary.

In conclusion, changes in CCT resulting from NaF strip intervention measured by the Pentacam should be considered during preoperative planning for LASIK, and clinicians are advised to avoid performing NaF strip application before obtaining precise corneal parameters.

The limitation in this study are results from age/gender matched controls were not included for comparison, and the effect of diurnal variations on the corneal pachymetry had not been investigated.

## Methods

Both eyes of participants scheduled to undergo LASIK were recruited from the Aier Eye Hospital of Wuhan University (Wuhan Aier Eye Hospital), Wuhan, China from January to March 2022Inclusion criteria for LASIK were as follows: (1) age ≥ 18 years; (2) a stable refraction for 2 years prior to surgery; (3) a minimum calculated residual corneal stromal bed thickness of ≥ 280 μm; (4) preoperative spherical error up to − 10.0 D and cylinder up to − 5.0 D^26^. All patients had a standard pre-operative evaluation including slit-lamp, and dilated fundal examinations; unaided distance visual acuity (UDVA), manifest refraction with corrected distance visual acuity (CDVA), and corneal topography (Pentacam)—using standardized reports for refractive surgery^27^. Patients with a history of ocular surgery or ocular trauma, concomitant active or previous ocular diseases, such as uveitis and glaucoma, and systemic diseases affecting wound healing, such as diabetes mellitus, collagen vascular diseases, or dry eye were excluded^28^. In addition, the subjects who was using medicals (topical and systemic) were excluded, such as hormone, and the patients who had used medicals more than 1 week were included. Participants were required to confirm that they had been awake for a minimum of 2 h to allow dissipation of overnight corneal edema^16^. In addition, the effect of contact lenses on corneal parameters was considered, and participants who had used soft and rigid contact lenses were requested to abstain from wearing their lenses for at least 7 days or 3 months before participation on the day of study^18^. Ethical approval (No.2022IRBKY02) was obtained from the Ethics Committee of Aier Eye Hospital of Wuhan University (Wuhan Aier Eye Hospital) prior to commencing the study. The study procedures were performed in accordance with the tenets of the Declaration of Helsinki, and written informed consent was obtained from all subjects.

The strips used in this study were 5.0 mm in width and 24.0 mm in length, and were impregnated with NaF (1.0 ~ 1.5 mg, Liaoning Meizilin Pharmaceutical Co., Ltd, China). Prior to use, the strip was wetted with a drop of sterile saline which is disposable,(10 ml. 90 mg, Tianjin King York Group Hubei Tianyao Pharmaceutical Co., Ltd, China), and excess NaF solution was shaken off gently and swiftly.

Then the examiners converted the lower eyelid with forefinger wearing sterile medical glove and the inferior palpebral conjunctiva came in contact with the strip swiftly and gently. After the treatment with NaF strip, the subjects were asked to blink three times, and then the broad beam of a slit lamp biomicroscope was used to observe the NaF-colored layer of the cornea. Normally, the BUT was more than 10 s, in patients in whom BUT was less than 5 s, dry eye was defined. NaF-positivity was defined as the part of the corneal epithelial defect that had yellow-green coloring^29^.

Topographic measurements of corneal parameters were consecutively obtained using a Pentacam before and 10 min after treatment with NaF strips in a dark room. Within 2 s, the system rotated 180°, acquiring 25 or 50 images, depending on the user settings^30^. Images that could not be processed by the devices, or that were incomplete, were discarded and immediately repeated until three valid measurements were obtained^24^. After collection of the data collection, the corneal parameters were reviewed by an examiner using the software supplied by the manufacturer.

## Statistical analysis

This study is a paired design before and after the intervention, and CCT was used as the main index of observation. According to the results of the preliminary experiment, the mean ± standard deviation of CCT is 8.82 ± 31.6. The sample size of 137 cases was calculated by PASS 15, and 172 sample were included considering the 20% dropout. All statistical analyses were carried out with SPSS 26.0 (SPSS Inc., Chicago, Illinois, USA), and GraphPad Prism 8.0 (GraphPad Software, San Diego, CA, USA) was used to draw the box plots. All data are expressed as the mean ± standard deviation (SD), and the level of statistical significance was set at *P* < 0.05. The normal distributions of all parameters wereconfirmed using the one-sample Kolmogorov–Smirnov test (*P* > 0.05). Paired samples t-test and Wilcoxon signed-rank test were used to compare normally and non-normally distributed data, respectively. The evaluations were conducted at a 95% confidence interval for the difference.

## Data availability

The datasets used and/or analysed during the current study available from the corresponding author on reasonable request.
